# PoreWalker: A Novel Tool for the Identification and Characterization of Channels in Transmembrane Proteins from Their Three-Dimensional Structure

**DOI:** 10.1371/journal.pcbi.1000440

**Published:** 2009-07-17

**Authors:** Marialuisa Pellegrini-Calace, Tim Maiwald, Janet M. Thornton

**Affiliations:** EMBL/EBI, The Wellcome Trust Genome Campus, Cambridge, United Kingdom; Max-Planck-Institut für Informatik, Germany

## Abstract

Transmembrane channel proteins play pivotal roles in maintaining the homeostasis and responsiveness of cells and the cross-membrane electrochemical gradient by mediating the transport of ions and molecules through biological membranes. Therefore, computational methods which, given a set of 3D coordinates, can automatically identify and describe channels in transmembrane proteins are key tools to provide insights into how they function.

Herein we present PoreWalker, a fully automated method, which detects and fully characterises channels in transmembrane proteins from their 3D structures. A stepwise procedure is followed in which the pore centre and pore axis are first identified and optimised using geometric criteria, and then the biggest and longest cavity through the channel is detected. Finally, pore features, including diameter profiles, pore-lining residues, size, shape and regularity of the pore are calculated, providing a quantitative and visual characterization of the channel. To illustrate the use of this tool, the method was applied to several structures of transmembrane channel proteins and was able to identify shape/size/residue features representative of specific channel families. The software is available as a web-based resource at http://www.ebi.ac.uk/thornton-srv/software/PoreWalker/.

## Author Summary

Transmembrane channel proteins are responsible for the transport of ions and molecules through biological membranes and are pivotal for the physiology of the cell. In fact, their incorrect functioning is involved or related to several diseases (diabetes, myotonia, Parkinson's disease, etc.). Moreover, their specificity and selectivity to different ions or molecules have been hypothesized and sometimes shown to strongly depend on the shape and size or amino acid composition of the channel. Therefore, computational methods to identify and quantitatively characterise channel geometry in transmembrane protein structures are key tools to better understand how they function. We have developed PoreWalker, a new method to detect and describe the geometry of these channels in transmembrane proteins from their 3D structures. The method is fully automated, very user-friendly, identifies the location of the channel and derives a number of channel features: diameter profiles at given heights along the channel, all the residues lining the channel walls, size, shape and regularity of the channel. These features can be very helpful in the study of how these channels might function. We have applied PoreWalker to several channel protein structures and were able to identify shape/size/residue features that were representative of specific channel families.

## Introduction

Transmembrane channel proteins play pivotal roles in maintaining the homeostasis and responsiveness of cells and the cross-membrane electrochemical gradient by mediating the transport of ions and molecules through biological membranes [1]. For instance, aquaporins facilitate the flux of water and small uncharged solutes across cellular membranes and, in humans, are involved in several diverse functions, like concentrating urine in kidneys and participating in forming biological fluids [2]–[5]. In contrast, potassium channels are fundamental regulators of cell membrane potential and control the action potential waveform and the secretion of hormones and neurotransmitters [6]–[8]. Moreover, a family of transmembrane proteins, known as translocons, have been found to mediate protein transfers between different cellular compartments and consequently to be involved in the folding of membrane and secretory proteins [9]. Understanding the structure and function of transmembrane channel proteins and studying their properties and biochemical mechanisms is therefore a very important task in biological and pharmaceutical research [10],[11].

Transmembrane channel proteins usually show a cavity spanning the whole protein, herein designated as the pore, which forms the path used by ions and/or molecules to cross the membrane. The pore has two openings, one on each side of the membrane, and it has been hypothesized (and in some cases shown) that the specificity and selectivity to different solutes is strongly dependent on the particular structural or amino acid composition features of the channel [5],[8],[12]. Consequently, computational methods for the identification and description of pores in transmembrane protein 3D-structures represent key tools to gain insights into how these proteins function.

To our knowledge, several methods for the analysis of protein surface and cavities have been developed [13]–[19] but the only currently available method for the structural analysis and visualisation of transmembrane channels is HOLE, developed in 1993 and still widely used [20],[21]. This elegant algorithm implements a Monte Carlo simulated annealing approach to find the path that a sphere of variable radius can use to go through a channel and also provides pore anisotropy analysis and conductance prediction tools. The path is optimised so that it can be considered as the route of a plastic sphere squeezing through the channel, i.e. at each point of the path the channel can accommodate the largest possible sphere. Three more recent methods, developed for the detection of internal cavities and tunnels in any protein structure, CAVER [22], its improved version MOLE [23] and MolAxis [24] can be applied to identify pores in transmembrane proteins. CAVER explores the protein inner space using a grid-based approach, while MOLE implements an algorithm based on Voronoi polyhedra. Both approaches use an optimality criterion based on the minimization of a cost function, which depends on reciprocal atomic distances, and calculates the optimal way out from a user-specified starting point inside the protein to the outside environment. MolAxis exploits computational geometry techniques, in particular the alpha shapes theory and the medial axis concept, to detect possible routes that small molecules or ions can take to pass through channels and cavities. It is worth highlighting here that all the four programs, to be applied to transmembrane proteins, require user-defined specific information about the geometry of the channel that necessitate a fairly good knowledge of the location of the pore and/or of key residues lining the pore walls, like a starting point for the path search through the channel or a vector approximating the location of the pore within the protein 3D-structure. Moreover, they provide only a limited description of the channel geometry mainly consisting of diameter values and some of the residues lining the pore walls.

Herein we present PoreWalker, a method to provide a detailed description of the three dimensional geometry of a channel (or pore) through a transmembrane protein, given the coordinates of the protein structure. These 3D pore descriptors provide a quantitative description, including the size, shape and regularity of the pore, which we hope will help to explain pore specificity, the critical biological function of these molecules. PoreWalker is fully automated, requiring only the 3D protein coordinates from the PDB file, and so can be applied to any new structure or across all transmembrane proteins in the PDB. The method was applied to several structures of transmembrane channel proteins and was able to identify shape/size/residue features representative of specific channel families. The software is implemented as a web-based resource at http://www.ebi.ac.uk/thornton-srv/software/PoreWalker/ and its source codes will soon be available upon request to the authors.

## Materials and Methods

The main goal of PoreWalker is to identify a channel in a transmembrane protein, through which a ligand (an ion or a small molecule) might pass. The channel can be defined by the pore-lining residues, which in most cases are accessible to solvent, and approximated by an axis, which ideally connects the two entrances of the pore and passes through the centre of the channel. The computational challenge is to identify the pore-lining residues from the protein 3D-structure and thus to define the axis and centre of the pore. Once this is done, the parameters defining the size and shape of the pore are easy to calculate.

The most direct approach to defining the channel would be to find the solvent accessible residues, and from these residues try to identify the pore-lining residues using geometric criteria. The most straightforward way to distinguish pore-lining residues from other accessible residues (on the outside of the protein) is to require that they ‘point towards’ the pore axis. However, since the latter cannot be defined without first knowing the pore-lining residues, it is necessary to adopt an iterative approach, whereby the axis is first approximated and then refined.

Ideally, the path taken by the ligand through the transmembrane protein will be linear and the pore will run approximately perpendicular to the plane of the membrane. Therefore, to make the first estimate of the channel axis, the algorithm takes into account the ‘special’ geometry of transmembrane proteins, in which the protein's secondary structures also tend to lie perpendicular to the transmembrane plane, running from one side of the membrane to the other. The channel axis is thus approximated as co-linear with these secondary structures and passing through their averaged centre of gravity.

In practice, paths can be convoluted and channels can be far from linear, as for the pores of some acid-sensing ion channels. Moreover, pores can be very narrow, with diameter values less than 1Å, so that pore-lining residues can not be straightforwardly detected as accessible to solvent. Therefore, the algorithm identifies a number of “local” pore centres at different pore heights (or slices) through the membrane so that the geometrically correct pore openings and path can be detected and a refined pore axis generated.

**Figure 1:**
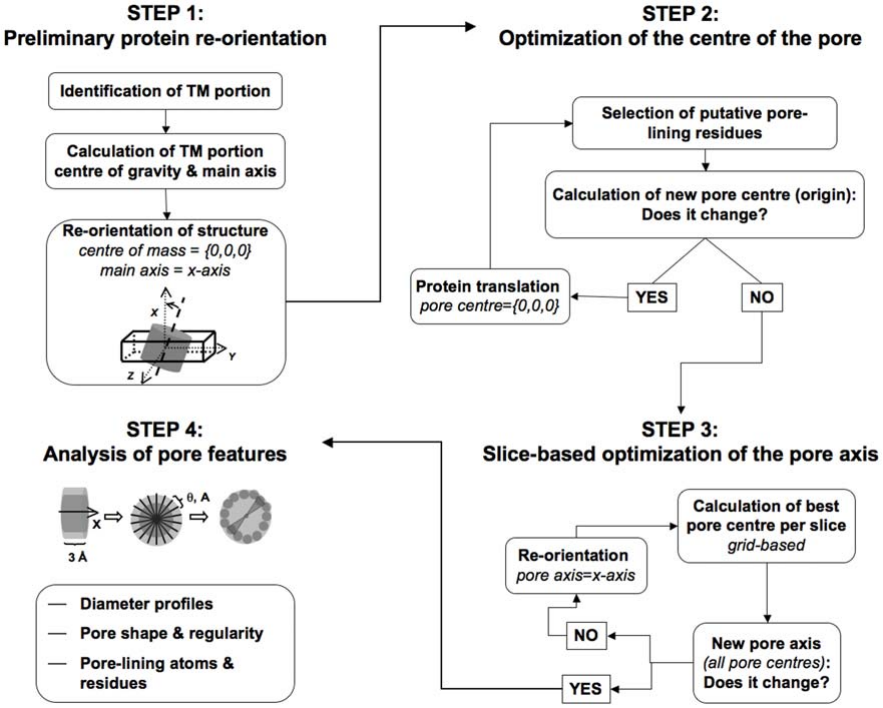
Flowchart of PoreWalker stepwise algorithm.

**Figure 2:**
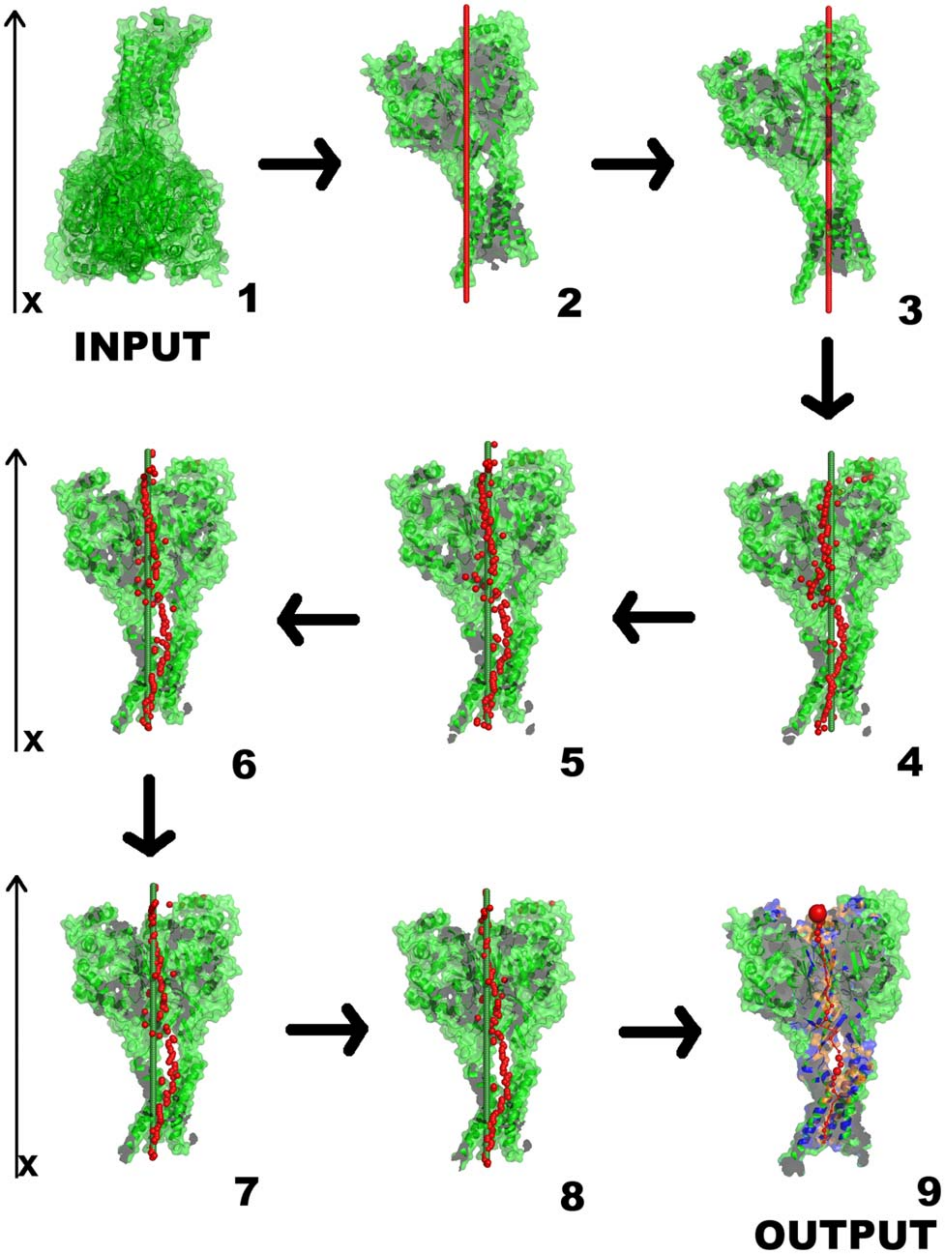
Changes of the pore axis during PoreWalker calculation for the ASIC1 acid-sensing ion channel (PDBcode 2qts [46]). Step 1: the 3D structure as submitted to the program. Step 2. Preliminary definition of the pore axis (shown in red). Step 3. Definition of pore centre and translation of the pore axis so that it passes through it (axis shown in red). Step 4–8. Iterative identification of local pore centres (red spheres) and variations in the position of the pore axis (green) during the refinement of the pore axis. Step 9. PoreWalker output.

The algorithm is heuristic and iterative, and includes the following steps (see Figure 1):

1. Definition of the channel axis as the average of the secondary structure vectors and passing through the centre of gravity of the protein;
2. Identification and maximization of the number of putative pore-lining residues and definition of the centre of the pore, as follows:
3.
  - use the current pore-axis to identify pore-lining residues as those which are accessible, close to the pore axis and whose C-alpha-C-beta vector points towards this axis;
  - use the detected pore-lining residues to redefine the centre of the pore;
  - iterate steps *a* and *b* until the number of pore-lining residues reaches a maximum.
    This defines a new geometrical ‘centre’ for the pore and a preliminary pore-axis, which is perpendicular to the plane of the membrane and passes through the pore centre;
4. Iterative refinement of pore axis. The protein structure is divided into slices (perpendicular to the current pore axis) and, for each slice, the local ‘centre’ of the pore, defined to generate a maximum diameter of the pore for that slice, is refined. These centres define the optimised ‘path’ of the cavity through the channel and are used to define the final pore axis. An example of how the pore axis changes during the iteration of steps 1 to 3 is shown in Figure 2;
5. Calculation of 3D descriptors defining the geometry and chemistry of the pore.

All programs included in the PoreWalker pipeline are developed in-house in C and PERL programming languages. The web-server is based on PERL-CGI protocol and the results of the four step calculations are summarised in pictures and text files displayed on the website and downloadable.

### 1. Preliminary definition of a pore axis and re-orientation of the protein

In transmembrane proteins, the channel runs approximately perpendicular to the membrane plane and parallel to the bundle or barrel that makes up the transmembrane portion of the pore. The first step of the program consists in re-orienting the protein structure so that the origin lies at the centre of gravity of the transmembrane portion of the protein and the bundle/barrel lies perpendicular to the membrane plane. The main axis of the transmembrane bundle/barrel is calculated according to the position of the secondary structure elements that putatively form it. Each secondary structure element in the protein is identified from the separation of sequential C-alphas as described in Supplementary Text S1 and, if the helix or the strand is longer than 15 or 10 amino acids, respectively, it is approximated by a vector, which starts at its centre of mass and points toward the centre of mass of the terminal four and two amino acids of the helix or strand, respectively. The length threshold was applied because, on average, transmembrane helices and strands used for this calculation need to be sufficiently long to cross the membrane. This excludes small helices which often do not lie perpendicular to the membrane plane. The sign of all the vectors is selected so that they point in approximately the same direction and the averaged vector is calculated. However, outlying secondary structures found to be more perpendicular than parallel to the bundle/barrel axis are excluded from the averaging at this stage so that the transmembrane portion of the structure is orientated as parallel to the membrane axis as possible.

The whole protein 3D structure is then re-oriented so that its calculated main axis overlaps with the x-axis of the current 3D system and the centre of gravity of its transmembrane portion lies at the origin. In this way, the structure is moved into a new reference frame that approximately aligns the transmembrane secondary structure elements perpendicular to the membrane. The pore axis is then approximated as coinciding with the protein main axis (see Figure 2, step 2). This starting assumption, despite its crudeness, simplifies and speeds up the following steps of the method.

### 2. Definition of the centre of the pore

The centre of the pore is defined by iteratively maximising the number of detected putative pore-lining residues, i.e. water-accessible amino acids pointing towards the pore axis. At the beginning of the process, the centre of the pore and the pore axis, i.e. the linear vector going through the middle of the pore, are assumed to correspond to the centre of mass of the protein and to the x-axis, respectively. Putative pore-lining amino acids around the pore axis are then selected to satisfy three criteria: (1) the relative sidechain solvent accessibility calculated by NACCESS ([25], downloadable at http://www.bioinf.manchester.ac.uk/naccess/) must be higher than 5%; (2) the vector defined by the C-alpha-C-beta bond must point towards the pore axis; and (3) the distance of the C-alpha atom from the pore axis must be below a given threshold. The distance threshold is calculated at each iteration as the smallest distance between any pore-lining residue C-alpha and the current pore axis plus 6 Å. This prevents the inclusion of “second shell” residues in the selection of putative pore-lining residues and in the calculation of the final centre of the pore. Glycines lack C-betas and are therefore treated differently. For each Gly, a dummy atom is defined as the average of 3D-coordinates of its backbone carbonyl carbon and amide nitrogen. This atom can be considered a mirror image of the C-betas of a virtual side chain located between the two hydrogen atoms bound to its C-alphas and can therefore be used to evaluate the orientation of Gly backbone atoms. Glycines with a total relative accessibility higher than 5% and with the dummy atom pointing away from the pore axis are defined as pore-lining.

A new centre of the pore is then calculated from the selected putative pore-lining amino acids and the protein structure is translated so that the new pore centre and the x-axis corresponds to the origin of the 3D-system and to the new pore vector, respectively.

The above procedure is performed iteratively and stops when the number of newly selected putative pore-lining residues converges to its maximum, indicating that the pore centre has reached its optimal position. As a result of this first process, the protein structure is translated in space so that the x-axis goes through the current best-guess of the centre of the pore and a preliminary list of putative pore-lining residues is generated (see Figure 2, step 3).

The effectiveness of this step of the method was assessed by monitoring the distance of the selected pore-lining residues from the pore centre, as described in Supplementary Text S1 and shown in Figure S1.

### 3. Refinement of pore axis and detection of cavity

To derive the best possible axis and cavity of the pore an iterative slice-based approach is used, in which the centre of the pore is systematically optimised for each slice and therefore eventual irregularities in the cavity can be detected. At each iteration, the protein structure is mapped onto a 3D-grid of 1Å steps and then sliced along the x-axis (i.e. the current pore axis) in 1Å thick layers. The pore centre of each slice is then identified by a grid-based approach so that it lies at the centre of the sphere with the maximum radius that the slice can accommodate. The maximum sphere and its centre are derived by expanding the sphere from the current centre until it clashes with a pore-lining atom, and systematically shifting the centre on the vertices of a 2D-grid so that the centre of the sphere of maximum volume for that slice can be identified.

The pore centre of the slice is initially set as the average of C-alpha and C-beta atoms of the putative pore-lining amino acids belonging to the slice selected in the previous step of the program, and the corresponding maximum sphere is calculated. A square 2D-grid perpendicular to the current pore axis (x-axis) is then built and used to optimize the location of the pore centre. The grid has 0.1 Å squares, it is centred at the pore centre, and its size depends on the sequence length of the protein and on the size of the pore. Grid vertices not surrounded by atoms in all the possible y and z directions are taken as located outside the pore and excluded from the optimization process. The sphere of maximum volume at a given centre is calculated by increasing its radius by 0.1 Å until it hits a vertex of the 3D-grid occupied by a backbone or C-beta atom. The current sphere radius is adjusted by subtraction of the atomic van der Waals radius (1.8 Å, corresponding to the average radius of all types of heavy atoms found in protein structures as in the AMBER united force field [26]) or approximate residue side chain radius (as in Levitt's amino acid ‘lollypop model’ [27]) if a backbone atom or a C-beta is hit, respectively. If the radius value is above any previously calculated radius, the current radius and corresponding sphere centre are taken as the maximum radius and pore centre for that slice.

At the end of the iteration, coordinates of the last four consecutive sphere centres at each end of the pore, that represent the two pore openings, are averaged to generate two points, which define the new pore axis. The structure is then re-oriented to align with the new vector (see Figure 2, steps 4–8). The last four consecutive spheres are used because the ends of the channels can be very irregular in term of shapes and therefore pore axes derived from the two very last sphere centres (one per end) often do not cross correctly one or both the pore entrances (the value 4 was derived on a trial-and-error basis in the range of values from 1 to 5).

The refinement process stops when the new pore vector “overlaps” to the old pore axis (i.e. when their angle is lower than 0.5 degrees) and the current pore axis and maximum sphere radii (i.e. those calculated in the previous iteration) are retained as optimal and used in the further analysis of the pore shape.

### 4. Analysis and prediction of pore features

The last step of the method is the analysis and calculation of three main pore descriptors: the pore-lining atoms and residues (Section 4.1), and the shape of the pore cavity (Section 4.2) and its regularity (Section 4.3).

#### 4.1 Identification of pore-lining atoms and residues

Pore-lining residues can be defined as amino acids contributing at least one atom to the inside surface of the pore. The pore surface can be considered as a continuum made up of horizontal and vertical layers of atoms. To investigate each layer, the protein structure can be cut horizontally through the pore axis into slices of thickness comparable to the diameter of a heavy atom (i.e. C, N or O), so that each slice in theory will include only one layer of atoms. Since the average diameter of protein heavy atoms in the AMBER force field is ∼3.6 Å, the slice size was approximated to its lower integer (3Å) so that the chance that two atoms from two adjacent layers are included in the same slice is minimised. Each slice can then be further divided into wedges (again of thickness comparable to the diameter of a heavy atom) so that, for each wedge, the atom closest to the pore centre can reasonably be considered as “the” pore-lining atom of that wedge. In particular, each 3Å slice is split into cylindrical segments (wedges, Figure 1-step 4) perpendicular to the pore axis and of a size dependent on the lowest distance (d_min_) between the pore centre and any atom belonging to that cylindrical segment and on the average diameter of a heavy atom. In this way, it can be assumed that each generated cylindrical segment includes only one pore-lining atom (see Supplementary Text S1), which will be the closest to the slice pore centre.

For each cylindrical segment, the lowest distance between the pore centre and any atom is then calculated and a first list of pore-lining atoms is derived. However, since pore sections included in slices are rarely circular, the wedge generation is repeated using the largest distance between the pore centre and any pore-lining residue (d_PLAmax_), so that more cylindrical segments are produced and all atoms within the anulus defined by circumferences of radii d_min_ and d_PLAmax_ can be detected as pore-lining. This decreases the chance of missing pore-lining residues because of irregularities in the pore shape.

#### 4.2 Description of the pore shape

**Figure 3:**
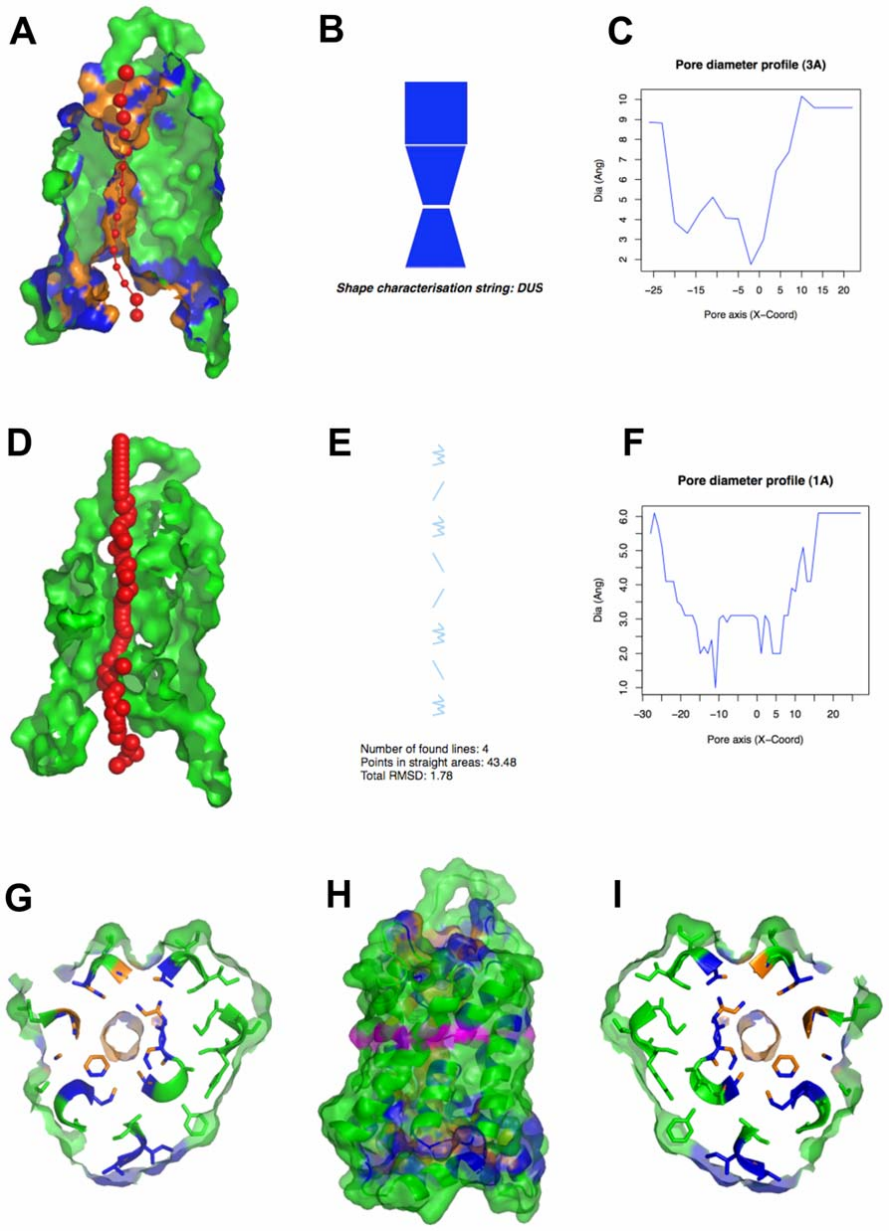
PoreWalker output for the bovine aquaporin-1 water channel (1j4n). (A) visualization of a pore section showing pore-lining residues and pore centres at 3Å steps: the section of the structure was obtained by cutting the protein structure along the xy-plane, where the x-axis corresponds to the pore-axis, and y-coordinate>0 only are displayed. Pore-lining atoms and residues are coloured in orange and blue, respectively. The remaining part of the protein is shown in green. Red spheres represent pore centres at given pore heights and their diameters correspond to 1/10 of the pore diameter calculated at that point; (B) representation of the shape of the pore and shape characterization string: the pore is simplified as a stack of building blocks going from the most negative to the most positive coordinate along the pore axis (x-axis). D, U and S indicate conical frustums of decreasing diameter, conical frustums of increasing diameter and cylinders, respectively; (C) pore diameter profile at 3Å steps corresponding the pore shape in (B). Pore axis (X-Coord): the position along the pore axis is shown as x-coordinate in Å. Dia (Ang): pore diameter value in Å; (D) visualization of a pore section showing the position of the biggest spheres (pore centres) that can be built along the channel at 1Å steps: the section of the structure was obtained by cutting the protein structure along the xy-plane. The protein structure is coloured in green; (E) diagram of the regularity of the pore cavity as number of lines that can approximate the positions of the pore centres at 1Å steps (PRINCIP). The pore is represented as a series of consecutive straight and wiggly lines representing channel areas where pore centres can (straight) or cannot (wiggly) be fitted to a line, going form the most negative to the most positive coordinate along the pore axis (x-axis). Vertical lines describe either the only low-RMSD areas throughout a pore or low-RMSD areas that are co-linear. Diagonal lines represent low-RMSD areas, which are different from the low-RMSD areas other identified along the channel. Curve lines indicate areas where pore centres are highly spread; (F) pore diameter profile at 1Å steps. Pore axis (X-Coord): the position along the pore axis is shown as x-coordinate in Å. Dia (Ang): pore diameter value in Å; (G)–(I) horizontal sections of the pore at the pore height highlighted in purple in (H) viewed from the bottom, i.e. the most negative coordinate along the pore axis (G) and from the top, i.e. the most positive coordinate along the pore axis (I).

The 3D shape of the pore (see Figure 3A) is simplified as a stack of two types of building blocks, truncated cones (i.e. conical frustums) and cylinders, so that a schematic “cartoon” diagram of the channel can be created. Building blocks are identified by analysing the trend of the smallest diameters calculated, along the whole pore, for each 3Å horizontal slice during the detection of pore-lining residues. Truncated cones are used to describe pore areas where diameter values vary linearly, while cylinders describe areas of constant diameter.

The variation of calculated slice diameters is analysed by linear fit of diameters within windows of variable width sliding across the sliced pore. Given a window, if the corresponding R correlation coefficient (R^2^) is lower than 0.5, the window is slid one slice further. Otherwise, the window is extended to the next slice diameter and a new R^2^ is calculated until R^2^ *_n+1_*<0.9 R^2^ *_n_*. The *n*- window is then taken as the best building block of the shape of the pore in this region.

Standard deviation (SD) values within windows giving positive linear fitting are used to decide if those pore areas approximate truncated cones or cylinders. As mentioned above, diameter values of truncated cones vary linearly from the lower to the upper circle, while they are constant in cylinders. The SD derived from a set of diameters approximating a cylinder will then be on average smaller (zero for a perfect cylinder) than the SD derived from diameters that approximate a truncated cone. Therefore, building block SD values above and below 1Å are taken to indicate truncated cones and cylinders, respectively. In addition, the type of a truncated cone (increasing or decreasing diameter) is detected from the difference between its first and last diameter.

To refine the description of the pore shape, the trend of calculated diameters is re-analysed using the same method but starting from the opposite end of the pore.

#### 4.3 Analysis of the regularity of the cavity

The regularity of the pore cavity is deduced from the positions of the pore centres calculated from the third step of the program, aimed at the optimization of the pore axis. If all the pore centres of a channel are co-linear, then the pore is linear and the cavity must be very regular and symmetric with respect to its axis. Otherwise, if only pore centres of certain areas of the channel are co-linear the pore may be symmetric but not linear and the cavity will be partly regular. If very few pore centres are co-linear, the cavity must be non-symmetric and the pore will be irregular.

Therefore, the deviation of the points modelling the pore centres from a straight line can reasonably be considered a measure of the linearity and regularity of the shape of the cavity. Linear areas along the pore are identified with a window-based approach similar to that described in section 4.2 from the 3D-coordinates of the pore centres calculated at 1Å slices (Section 3) and are defined as segments of the pore with an RMSD≤0.5 Å. RMSD values are calculated by PRINCIP, a program included in the SURFNET package [18]). Ideally, a very regular pore will have shape varying symmetrically with respect to the pore axis and will show the pore centres calculated at various depths along the cavity lying on a straight line, which passes through the centre of the pore.

## Results/Discussion

### Output web-page

Pore descriptors calculated by PoreWalker for a submitted structure are summarised in the corresponding output webpage, which shows the features of the channel cavity and several visualizations of the pore based on the identified pore-lining residues. As an example, the output of the bovine aquaporin-1 (PDB code 1j4n) is summarised in Figure 3. The 3D shape of the pore is simplified in 2D as a stack of building blocks shaped as trapezia for funnel-like shapes (Figure 3B) going from the most negative to the most positive coordinate along the pore axis. In addition, the pore cavity is represented as a series of consecutive straight and wiggly lines representing channel areas where pore centres can (straight) or cannot (wiggly) be fitted to a line, respectively (Figure 3E). It is worth highlighting here that the approach does not take into account any chemistry (e.g. H-bonds) but just calculates the path of the pore centres. In practice, ions/molecules may well hop between low energy off-centre sites, within the channel, that optimize their interactions with pore residues during their passage through the channel.

Vertical and horizontal visualizations of the pore help to provide a better understanding of the channel features. Vertical sections (Figure 3A,D) are generated halving the protein structure along the pore axis, while horizontal sections (Figure 3G,I) are produced as 5Å slices of the protein structure perpendicular to the pore axis. Amino acids are coloured according to whether they are classified as pore-lining and red spheres represent optimal pore centres.

### Tests on experimental structures and comparison with other methods

**Figure 4:**
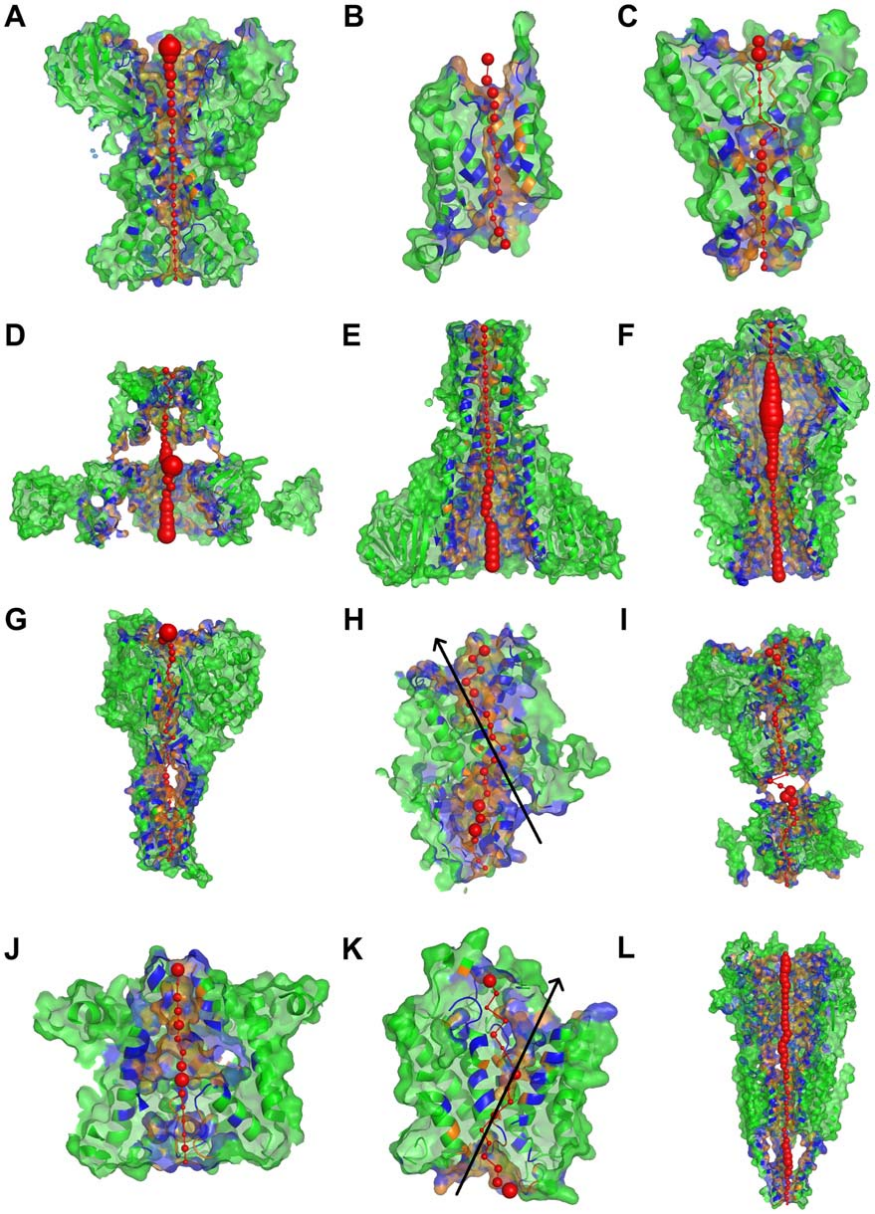
PoreWalker visual representations. Images show xz-plane sections, z-coordinate>0 only, with the x-axis corresponding to the pore axis. (A) KirBac1.1 potassium channel (1p7b); (B) bovine aquaporin-1 (1j4n); (C) KcsA potassium channel (1k4c); (D) MthK calcium-gated potassium channel (1lnq); (E) CorA Mg^2+^ transporter (2iub); (F) MscS voltage-modulated mechanosensitive channel (2oau); (G) ASIC1 acid-sensing ion channel (2qts); (H) SecYE-beta protein-conducting channel (2yxr); (I) Kv1.2 voltage-gated potassium channel (2a79); (J) sodium-potassium channel (2ahy); (K) Amt-1 ammonium channel (2b2f); (L) nicotinic acetylcholine receptor (2bg9). Pore-lining atoms and residues are coloured in orange and blue, respectively. The rest of the protein is shown in green. Red spheres indicate pore centres at 3Å steps and their size is proportional to the pore diameter in that point. Black arrows indicate the main pore axis as identified by visual analysis of the structure.

**Figure 5:**
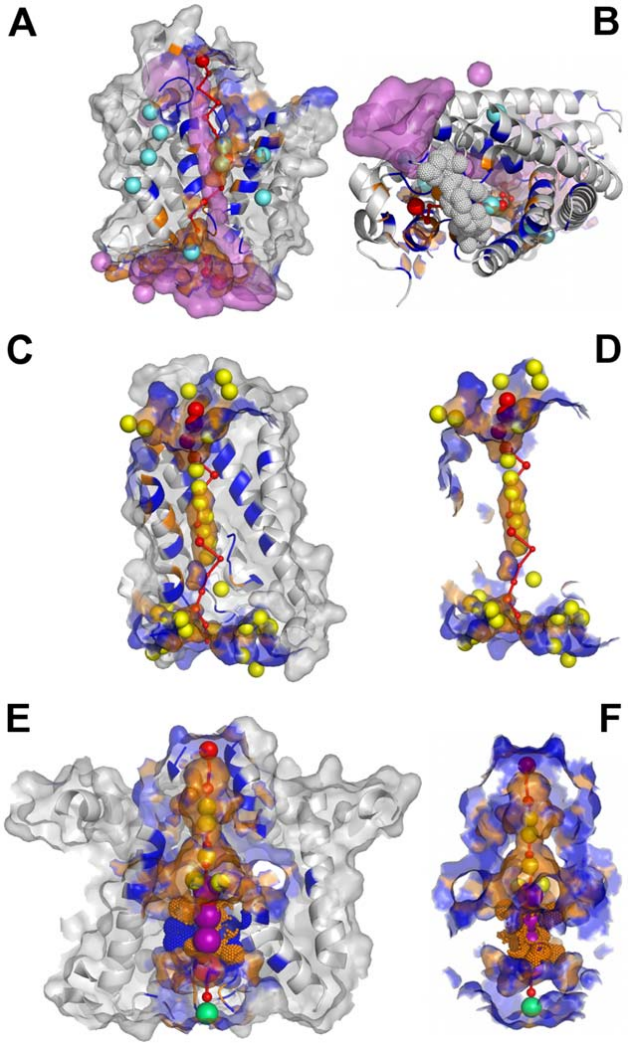
PoreWalker results and the molecules of solute found in the 3D structures. (A)–(B) PoreWalker and HOLE results obtained for the Amt-1 ammonium channel (2b2f). (A)-Vertical view. PoreWalker cavity is shown as y-coordinate>0 only of the protein section along the xy-plane, where the x-axis corresponds to the pore axis. Pore-lining atoms and residues are coloured in orange and blue, respectively, and the rest of the protein is shown in light grey. Red spheres represent pore centres at 3Å steps and their size is proportional to the pore diameter at that point. HOLE cavity is shown as purple surface. Xenon atoms, indicating the presence of cavities within the protein structure, are shown in light blue. (B)-Top view. The colour scheme is as in (A). White dots indicate the external loop, which divides the channel top gate into multiple exits. (C)–(F)-Comparison of PoreWalker results with the actual position of solute molecules for the SoPiP2;1 water channel (C–D, 1z98) and the sodium potassium channel (E–F, 2ahy). Pore visualizations follow the colour scheme in (A). Water molecules are shown in yellow, Na^+^ ions in purple and Ca^2+^ ions in green. (C) and (E) display PoreWalker sections as in (A). (D) and (F) show the pore only, represented as surface of the pore-lining atoms and residues only.

PoreWalker was tested on the 19 structures from the “Membrane Proteins of Known 3D Structure” resource (http://blanco.biomol.uci.edu/Membrane_Proteins_xtal.html) listed in Table 1, that include both ion and small molecule channels with straight and curve pores. Results are shown in Table 1, Figure 4 and Supplementary Figure S2. Although there is no fully comprehensive experimental data to assign with certainty the location and residue composition of channels in transmembrane protein 3D-structures, the position of the pore axis and of the pore centres, visually analysed in relation to the protein structure, and the minimum diameter value give a hint of the effectiveness of the method. From visual inspection, PoreWalker seems able to locate correctly the pore axis and the pore centres in most of the cases and therefore to identify fairly correctly the amino acids that line the pore walls with one or more atoms. In fact, the pore axis seems wrongly located only for the Amt-B (Figure 4K), Amt-1 (Supplementary Figure S2E) and the SecYE-beta translocon (Figure 4H) channels (PDB codes 1xqf, 2b2f and 2yxr, respectively). Both Amt-B and Amt-1 channels share a common hour-glassed shape with multiple exits at one of the pore gates and can therefore be thought to include more than one transmembrane tunnel of different length (Figure 5B). Likewise, the SecY-beta translocon shows two flexible loops at both sides of the channel that make a further narrower but longer cavity crossing the protein structure. Despite the misassignments of pore axis and pore centres, in these three examples most of the pore-lining residues still seem to be identified correctly because the calculated optimal cavities, indicated by red spheres, partially overlap with the “true” cavities, indicated by the black arrows.

**Table 1:** List of protein structures in the test set.

PDBid	Protein name	Res^a^	Ref	Shape String^b^	% centres in lines^c^	D(min)	D(min)	D(min)	D(min)	R^2^ *(PW-HOLE)* d
						*PW*-3Å	*MolAxis*	*PW*-1Å	*HOLE (1Å)*	
1k4c	KCSA potassium channel	2.00	[30]	DUDU	91.53	0.922	0.611	0.500	0.681	0.740
1lnq	MTHK calcium-gated potassium channel	3.30	[33]	DUD	55.41	0.315	1.128	1.000	−0.046	0.958
1p7b	KirBac1.1 inward-rectifier potassium channel	3.65	[34]	SUDSUDSU	100.00	1.110	0.709	1.100	0.508	0.918
1xl4	KirBac3.1 inward-rectifier potassium channel	2.6	n.p.	DUDUSDU	96.47	0.872	NF	1.000	0.512	0.925
1xqf	AmtB ammonium channel	1.8	[35]	UDUD	58.14	0.619	NF	1.000	−0.075	0.750
2a79	Shaker Kv1.2 potassium channel	2.9	[36]	UDUDSUDSUD	49.24	0.830	NF	1.000	0.981	0.583
2ahy	Sodium-potassium channel	2.8	[37]	SDUD	100.00	0.700	NF	0.500	−0.018	0.127
2b2f	Amt-1 ammonium channel	1.54	[38]	DSUS	27.45	0.575	NF	1.000	−0.098	0.017
1ymg	AQP0 water channel	1.9	[39]	DSD	78.57	1.631	NF	2.000	0.781	0.814
1j4n	AQP1 water channel	2.2	[40]	DUS	43.48	1.752	NF	1.000	0.997	0.615
1z98	SoPIP2;1 plant water channel	2.1	[41]	SDUD	29.55	0.724	NF	1.000	0.191	0.000
2bg9	Nicotinic Ach receptor	4.0	[42]	UDSUS	80.00	4.872	NF	3.099	2.087	0.814
2iub	CorA Mg2+ channel	2.9	[43]	DSD	85.71	3.466	1.580	3.099	1.247	0.834
2oar	MscL mechanosensitive channel	3.5	[44]	SDUDU	85.88	1.663	0.791	3.099	0.927	0.956
2oau	MscS voltage-modulated mechanosensitive channel	3.7	[44]	DUD	90.43	3.595	2.629	4.099	1.674	0.951
2qks	Kir3.1 prokaryotic Kir potassium channel	2.2	[45]	DUDUDS	100.00	0.629	0.464	1.000	0.281	0.817
2qts	ASIC1 acid-sensing ion channel	1.9	[46]	UDUDUD	42.73	0.275	0.630	1.000	−0.029	0.450
2vl0	pLGIC pentameric ligand-gated ion channel	3.3	[47]	DUDSUDSU	89.11	2.019	1.153	3.099	0.956	0.776
2yxr	SecYE-beta protein conducting channel	3.6	[48]	USDUS	32.20	2.854	0.572	1.000	0.079	0.095

a **Res**: atomic resolution of the protein structure in Å.

b **Shape String**: shape identified by PoreWalker, going from the lowest to the highest coordinate along the pore axis. D, U and S indicate decreasing diameter conical frustum, increasing diameter conical frustum and cylinder, respectively.

c **% centres in lines**: percentage of pore centres at 1Å step that have been identified as part of one or more lines by PoreWalker.

d **R^2^ (PW-Hole)**: correlation of diameter values calculated by HOLE and PoreWalker at 1Å step.

In terms of pore shape, PoreWalker seems to recognise common sub-shapes across channel families. For instance, all aquaglyceroporins show a DU-like string shape (where D and U represent funnel-like shape of decreasing and increasing diameter, respectively), which represents a hour-glasssed shape confirmed by a few published data [5],[28],[29]. Likewise, potassium channels present a shared sub-shape, a DUD sub-string shape at the cytoplasmic side of the channel, that is in agreement with the channel features reported by Mackinnon et al., i.e. a constriction at the cytoplasmic side, the internal pore, widening into a larger water-filled void, the internal cavity, which leads towards the narrow selectivity filter located at the periplasmic side of the channel [12]. In addition, the linearity of the cavity seems to give some insights on the pore selectivity to different types of solutes (Table 1). In fact, 10 of the 13 channels for inorganic ions in the set showed a very regular cavity, with average percentage of co-linear pore centres of 91.9% (SD = 7.0%) and organic small molecule/ion channels had less regular pores, with percentage of co-linear centres lower than 60%.

**Figure 6:**
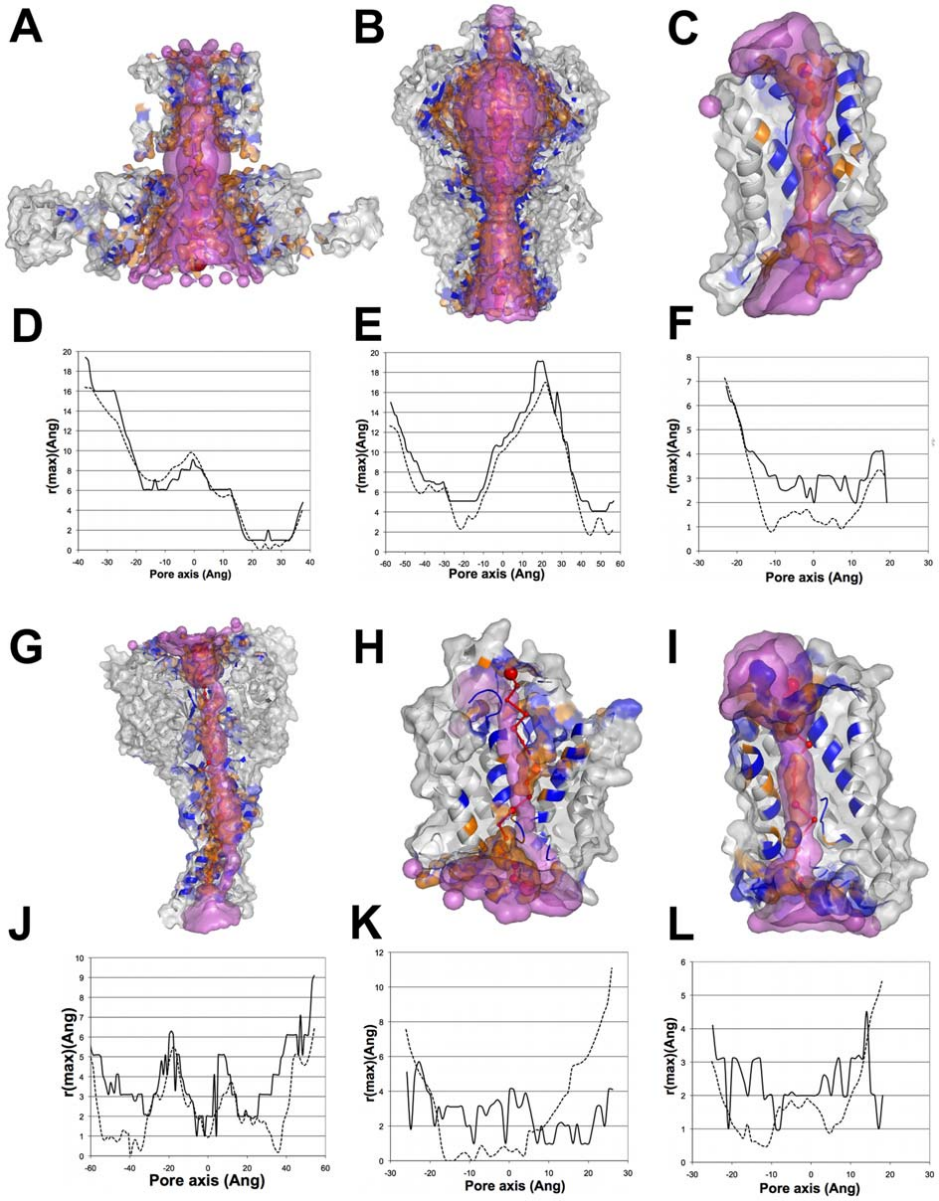
Comparisons of channel cavities identified by PoreWalker and HOLE and corresponding 1Å step profile diameters. (A)&(D) MthK potassium channel (1lnq), (B)&(E) MscS voltage-modulated mechanosensitive channel (2oau); (C)&(F) bovine aquaporin-0 (1ymg), (G)&(J) ASIC1 acid-sensing ion channel (2qts); (H)&(K) Amt-1 ammonium channel (2b2f); (I)&(L) SoPiP2;1 water channel (1z98). PoreWalker cavities are shown as y-coordinate>0 only of the protein section along the xy-plane, where the x-axis corresponds to the pore axis. Pore-lining atoms and residues are coloured in orange and blue, respectively, and the rest of the protein is shown in light grey. Red spheres represent pore centres at 3Å steps and their size is proportional to the pore diameter at that point. HOLE cavities are shown as purple surface. PoreWalker and HOLE profile diameters are shown as solid and dotted lines, respectively. Corresponding R^2^ values are 0.958, 0.951, 0.814, 0.450, 0.017 and 0.000 for 1lnq, 2oau, 1ymg, 2qts, 2b2f and 1z98, respectively.

For completeness, PoreWalker output was also compared with results obtained using HOLE and MolAxis on the same set of structures. A systematic comparison with MOLE results could not be performed because, probably due to the intrinsic looseness of some structures, like the MthK and the ASIC1 channels, many of the tunnels identified by MOLE lie parallel and not perpendicular to the membrane axis and could not be considered as transmembrane. Within the set of pore features produced by PoreWalker and HOLE, the only comparable quantitative measure is the diameter, calculated along the pore at given heights. Diameter profiles obtained at 1Å steps for the 19 transmembrane proteins in the set were compared using the R^2^ correlation coefficient (see Supplementary Text S1, Table 1, and Figures 6, S3, S4 and S5). Pore diameter analyses performed with the two methods showed good agreement for 12 of the 19 diameter profiles, with R^2^ higher than 0.75. However, the remaining 7 profiles showed very poor correlation coefficients, with R^2^ very close or equal to zero. This behaviour seemed to be strongly affected by the regularity of the cavity. In fact, R^2^ values showed a good correlation with the number of co-linear pore centres (Supplementary Figure S5) with a R^2^ of 0.70 and only one strong outlier, the sodium-potassium channel (PDB code 2ahy). The disagreement between the two profiles in this case was due to a completely different pore exit at the top channel side identified by HOLE that seems visually incorrect and makes the diameter trend in that area very peculiar.

As for MolAxis, the program does not calculate diameter values at given heights along the channel axis but provide a partial list of the amino acids that contribute to the pore surface. Therefore, minimum diameters and pore lining residues were used to compare PoreWalker and MolAxis results. MolAxis could not identify a channel for 9 of the 19 test protein structures (Table 1), the water, glycerol and ammonia channels and three potassium channels. For the remaining 10 proteins, minimum diameter values derived from the two methods gave poor correlation (R^2^ = 0.46). The exclusion of the SecYE-beta translocon, incorrectly characterised by PoreWalker, lead to an R^2^ of 0.69 (corresponding MolAxis-HOLE R^2^ were 0.60 and 0.57, respectively). Minimum diameters calculated by HOLE and PoreWalker gave a better correlation, with R^2^ of 0.54 and 0.90, respectively (the overall R^2^ on the 19 structure set was 0.67). In term of pore-lining residues, MolAxis provides a list of the amino acids responsible for the calculated diameters, i.e. a subset of the amino acids that make the surface pore. MolAxis pore-lining residues were fully included in PoreWalker pore-lining residue list in all the compared proteins but the SecYE-beta translocon. In this case, 23 of the 24 pore-lining residues detected by MolAxis were included in the list generated by PoreWalker, showing that the method can reliably identify amino acids which build a channel despite mis-placements of its pore vector.

Finally, transmembrane pores identified by PoreWalker were found to coincide well with molecules of solute found in the 3D structure. Figure 5C–F shows the SoPIP2;1 plant aquaporin (1z98) and the sodium-potassium channel (2ahy) filled with water molecules and sodium and calcium cations, respectively. In both cases the cavities generated by PoreWalker completely surround and include water molecules and ions, which provide good evidence for the location and shape of the pore. Interestingly, PoreWalker is also able to identify the two main choke points in the water channel of the SoPIP2;1 reported to be in a closed state -the canonical Ar/R constriction site near the top of the pore and a narrower restriction close to the bottom of the channel (Figure 5D). The method can therefore analyse and characterise both “open” and “closed” transmembrane protein channels and transmembrane transporters.

### An example case: the KcsA potassium channel

The KcsA potassium channel is a homotetrameric integral membrane protein with high sequence similarity to all the potassium channels, particularly in the pore region. Its channel includes three elements: 1) a narrow entrance, known as the internal pore, starting at the intracellular side of the membrane; 2) an internal cavity, about 10Å in diameter, at the middle of the membrane; 3) a further narrowing, the selectivity filter, which leads to the extracellular environment [30]. The KcsA channel is therefore a good target to assess the ability of PoreWalker to detect constrictions, gates and internal cavities in the 3D-structure of a channel protein.

**Figure 7:**
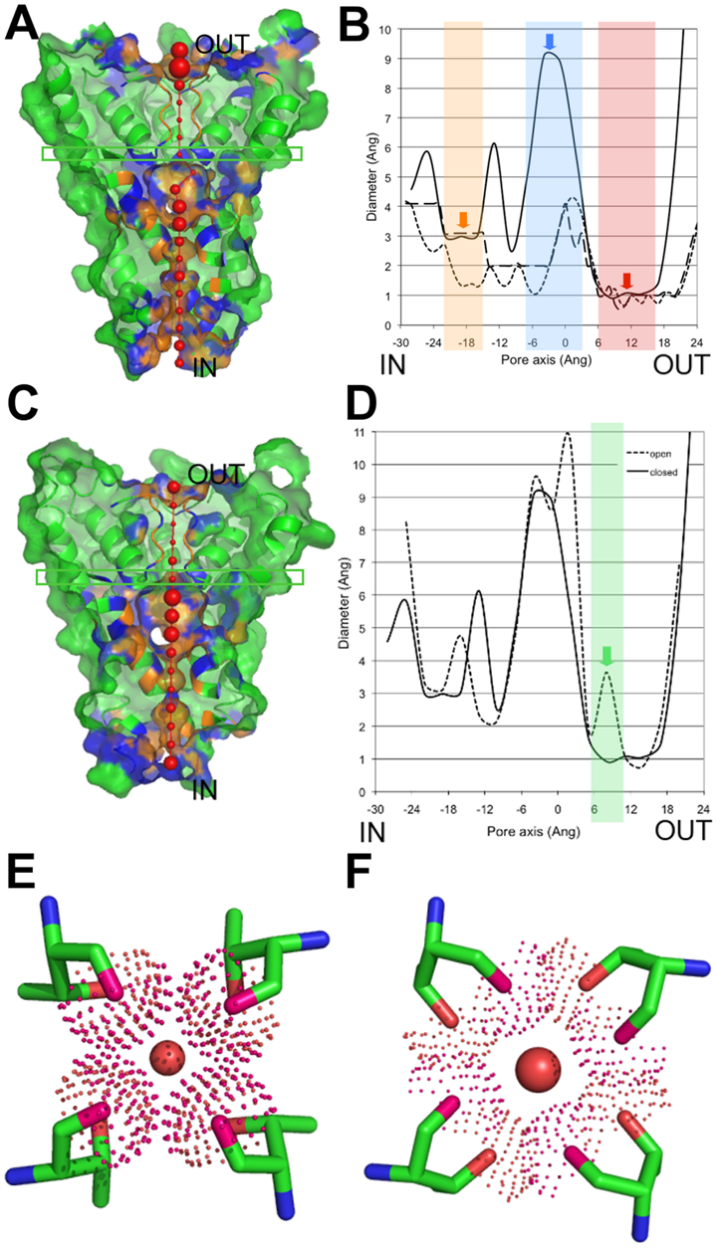
PoreWalker results for the 3D-structures of the KcsA K^+^ channel at low (1k4c) and high (1bl8) concentration of K^+^. (A),(C)-PoreWalker visual representation (xz-plane section, z-coordinate>0 only, x-axis corresponding to the pore axis of KcsA K^+^ channel at low (1k4c, A) and high (1bl8, C) concentration of K^+^. Pore-lining atoms and residues are coloured in orange and blue, respectively, and the rest of the protein is shown in green. Red spheres represent pore centres at 3Å steps and their size is proportional to the pore diameter at that point. IN and OUT indicate the cytoplasmic and periplasmic side of the pore, respectively. (B)-Diameter profiles calculated by PoreWalker standard protocol (3Å steps, solid line), HOLE (1Å steps, dotted line) and PoreWalker-1Å steps (dashed line). The internal pore, the internal cavity and the selectivity filter are highlighted in orange, blue and red, respectively. (D)-Diameter profiles calculated by PoreWalker standard protocol for the low-K^+^ (solid line) and high-K^+^ (dotted line) KcsaA channel structures. The entrance of the selectivity filter is shown in green and is found at the pore height highlighted by a green box in the channel structures (A for the low-K^+^ channel and C for the high-K^+^ channel). (E)–(F) Different conformation of the Thr75s lining the entrance of the selectivity filter in the low-K^+^ (E) and high-K^+^ (F) channels. Backbone and sidechain oxygens are coloured in dark pink and red, respectively, and their atomic volume is shown by dots. Red spheres represent the pore centres at the entrance of the selectivity filter and their size is proportional to the pore diameter predicted for that point (0.92Å and 3.63Å for the low-K+ (E) and high-K+ (F) channels, respectively).

The 3D structures of the Kcsa potassium channel in the presence of low (3 mM, Figure 7A) and high (200 mM, Figure 7C) K^+^ concentrations are available at the wwPDB (codes 1k4c [30] and 1bl8 [8], respectively) and their pore features were derived and analysed using PoreWalker (Figures 7–9). The diameter profile of the low-K^+^ channel (Figure 7B, solid line) shows that PoreWalker can neatly identify the three main features of the channel: first a ∼3Å narrowing corresponding to the internal pore, the internal ∼9.0Å bigger cavity and a second narrower (∼1Å) constriction corresponding to the selectivity filter, highlighted in the Figure in orange, blue and red, respectively. It is interesting to notice here that diameter values calculated at 1Å steps by both HOLE (dotted line) and PoreWalker (dashed line) at the maximum width of the internal cavity (∼4Å) were significantly smaller than those reported in the description of the 3D-structure [30] (∼10Å) and found using the standard PoreWalker protocol at 3Å steps (∼9Å).

**Figure 8 pcbi-1000440-g008:**
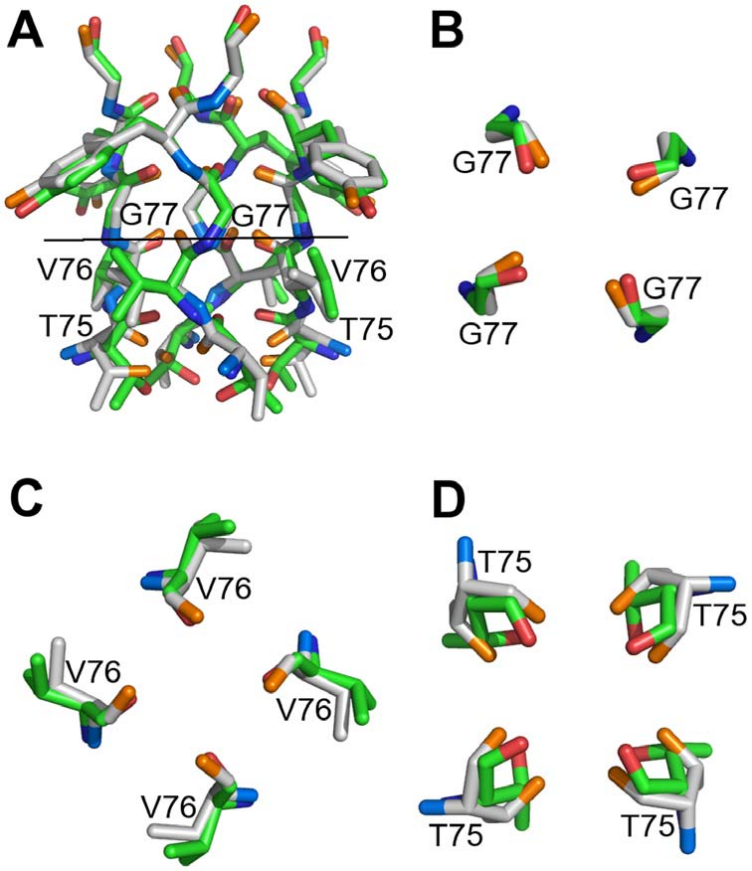
Superpositions of the selectivity filter 3D-structures of the low-K^+^ (green/red/blue colour scheme) and high-K^+^ (white/orange/blue colour scheme) KcsA channels. (A) the whole filter (C-alpha RMSD = 0.38Å, all atom RMSD = 0.58Å), (B) G77s only, top view (all atom RMSD = 0.33Å), (C) V76s only, top view (all atom RMSD = 0.58Å), (D) T75s only, top view (all atom RMSD = 0.99Å).

**Figure 9:**
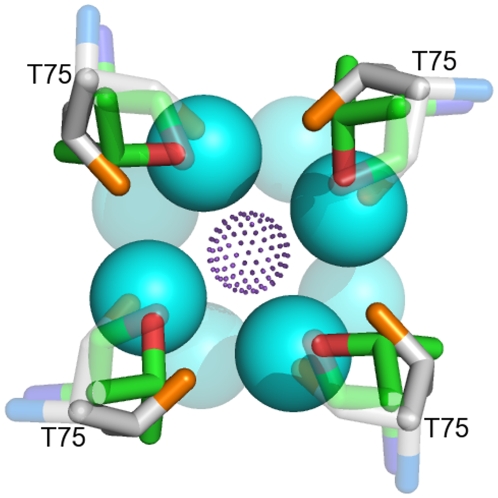
Superposition of the 3D-structures of Thr75s from the low-K^+^ (green/red/blue colour scheme) and high-K^+^ (white/orange/blue colour scheme) KcsA channels, top view. Water molecules from the low-K^+^ structure are shown in light blue. Violet dots represent a K+ ion. Thr side chain atoms and the four water molecules interacting with them are shown as opaque. Thr backbone atoms and the remaining four water molecules of the K^+^ hydration shell are shown as transparent.

The calculated diameters of the internal pore and cavity also strongly agree with the proposed mechanism of ion conductance through the pore. In fact, potassium cations are thought to move through the internal pore and cavity in a hydrated form and to be dehydrated at the selectivity filter. The internal pore detected by PoreWalker is ∼3Å in diameter and could allow through one water molecule per time (the average diameter of a water molecule is usually taken as 2.8Å). Therefore, K^+^ ion could move through it alternating with water molecules. On the other side, the selectivity filter has a predicted diameter of ∼1Å and could therefore let through only dehydrated K^+^ cations.

The comparison of the diameter profiles of the channel in presence of low and high quantity of ions (Figure 7D, solid and dotted line, respectively) showed that besides expected differences at the cytoplasmic side of the pore, where a gate mechanism is known to operate, the entrance of the selectivity filter is ∼2.5Å wider at high concentrations of K^+^. According to PoreWalker, the pore lining residues, which define access to the selectivity filter, are the Thr75s from the four chains making up the pore. The difference in pore diameters at this point seems mainly to be due to different Thr sidechain conformations (Figure 7E–F). A significant difference in the two conformations of the KcsA selectivity filter had been previously highlighted at the level of residues Val76 and Gly77. A deeper analysis of the whole selectivity filter (Figure 8A) showed that the periplasmic side of the filter (at the top of the Figure) varies very slightly, while a major change is hinged at Gly77 and extends through Val76 to Thr75, where a pincher-like shutting mechanism could reasonably be hypothesized (RMSDs of all-atom superpositions were 0.33Å, 0.58Å and 0.99Å for Gly77 (Figure 8B), Val76 (Figure 8C) and Thr75 (Figure 8D), respectively). Besides, the internal cavity accommodates K^+^ ions as hydrated by eight water molecules. The 3D-structure of the low-K+ channel cavity (Figure 9) shows that the four water molecules facing the filter are aligned to the sidechain oxygens of Thr75s and can make hydrogen bonds with them (inter-oxygen distances are 3.9Å). Moreover, their distances from the corresponding K^+^ ion are close to optimal (3.4Å versus 2.8Å [31]). Therefore, it might be reasonably thought that the pinching mechanism could be aimed at weakening the water-K+ hydration complex by increasing the distance between the water molecules and the ion to facilitate its way into the pore.

### Conclusions

We developed PoreWalker, a novel web-available method for the detection and characterisation of channels in transmembrane proteins from their three-dimensional structure. PoreWalker is fully automated and very user-friendly, requiring as input only the 3D coordinates of a transmembrane protein structure. A key prerequisite of the submitted structure is the presence of a transmembrane helix bundle or beta-barrel creating the pore, which is needed for the geometrical identification of the main protein axis. If this condition is not met, the detection/description cannot be performed with the current version of the software.

In term of outputs, in addition to diameter profiles, PoreWalker describes several specific pore features, in particular the shape and the regularity of the channel cavity, the atoms and corresponding amino acids lining the pore wall, and the position of pore centres along the channel. These features can be very helpful to gain further insights into the functional and structural properties of transmembrane protein channels by triggering specific *in silico* or experimental analyses, as shown from the recent characterization of the bacterial TolC channel [32].

PoreWalker is based on the assumption that, in a transmembrane channel protein, the pore is made by the longest cavity crossing the protein along the main axis of its transmembrane portion and therefore detects the longest widest cavity in a transmembrane protein structure. However, there are cases, as in the Amt-B and the SecYE-beta translocon, where the longest widest cavity does not correspond to the most likely “true” channel and in such cases the method assigns incorrectly one or both the pore gates. Interestingly, for these examples, calculated optimal cavities partially overlapped with the “true” cavities and most of the pore-lining residues were anyway identified properly.

In summary, PoreWalker provides a robust and automated resource to interpret, coordinate data and derive quantitative descriptors, which help to provide a deeper understanding and classification of membrane protein structures.

## Supporting Information

Figure S1 Trends of coefficient of variation (CV) normalised according to the distance threshold used to detect putative pore-lining residues (CV/D) and to the number of detected putative pore-lining residues (CV/N(aa)).
(0.61 MB DOC)

Figure S2 PoreWalker visual representations. Images show xz-plane sections, z-coordinate>0 only, with the x-axis corresponding to the pore axis. The remaining protein structures in Table 1 are shown. (A) KirBac3.1 potassium channel (1xl4); (B) MscS voltage-modulated mechanosensitive channel (2oar); (C) bovine aquaporin-0 (1ymg); (D) Kir3.1 prokaryotic Kir potassium channel (2qks); (E) Amt-B ammonium channel (1xqf); (F) pLGIC pentameric ligand-gated ion channel (2vl0); (G) plant SoPIP2;1 water channel (1z98).
(3.62 MB TIF)

Figure S3 PoreWalker and HOLE diameter profiles at 1Å steps. Solid and dotted lines indicate PoreWalker and HOLE diameter profiles, respectively. (A) KirBac1.1 inward-rectifier potassium channel (1p7b, R2 = 0.918); (B) bovine aquaporin-0 (1j4n, R2 = 0.615); (C) KcsA potassium channel (1k4c, R2 = 0.740); (D) MthK calcium gated potassium channel (1lnq, R2 = 0.958); (E) KirBac3.1 inward-rectifier potassium channel (1xl4, R2 = 0.925); (F) Amt-B ammonium channel (1xqf, R2 = 0.750); (G) bovine aquaporin-0 (1ymg, R2 = 0.814); (H) plant SoPip2;1 water channel (1z98, R2 = 0.000); (I) shaker Kv1.2potassium channel (2a79, R2 = 0.583); (J) sodium-potassium channel (2b2f, R2 = 0.017); (K) Amt-1 ammonium channel (1p7b, R2 = 0.918); (L) nicotinic acetylcholine receptor (2bg9, R2 = 0.814).
(0.93 MB TIF)

Figure S4 PoreWalker and HOLE diameter profiles at 1Å steps. Solid and dotted lines indicate PoreWalker and Hole diameter profiles, respectively. (A) CorA Mg2+ channel (2iub, R2 = 0.834); (B) MscL mechanosensitive channel (2oar, R2 = 0.956); (C) MscS mechanosensitive channel (2oau, R2 = 0.951); (D) Kir3.1 prokaryotic Kir potassium channel (2qks, R2 = 0.817); (E) ASIC1 acid-sensing ion channel (2qts, R2 = 0.450); (F) pLGIC pentameric ligand-gated ion channel (2vl0, R2 = 0.776); (G) SecYE-beta protein conducting channel (2yxr, R2 = 0.095).
(0.63 MB TIF)

Figure S5 Diameter profiles *versus* linearity of the cavity. The correlation between R2 values of PoreWalker-HOLE diameter profiles and the percentage of number of pore centres at 1Å steps that can be fit on one or more lines with PRINCIP is shown. Each point represents one protein. The starred point indicates the only outlier point (sodium-potassium channel, PDBcode 2ahy).
(0.12 MB TIF)

Text S1 Supplementary text.
(0.12 MB DOC)
