# Supplementary material for: PoreWalker: A Novel Tool for the Identification and Characterization of Channels in Transmembrane Proteins from Their Three-Dimensional Structure
Source: PLoS Comput Biol. 2009 Jul 17;5(7):e1000440. doi: 10.1371/journal.pcbi.1000440 (PMC2704872; doi:10.1371/journal.pcbi.1000440)
Supplement: Figure S1 — Trends of coefficient of variation (CV) normalised according to the distance threshold used to detect putative pore-lining residues (CV/D) and to the number of detected putative pore-lining residues (CV/N(aa)). (0.61 MB DOC) [file pcbi.1000440.s001.doc]

**Supplementary Text S1**

**Geometry-based detection of secondary structure elements.**

Secondary structure elements in the structure are identified from distances between C-alpha atoms. In particular, if the distance between the C-alpha of an amino acid *n* and the C-alphas of amino acids *n*-3 and *n*+3 is between 4.25 and 6.75 Å, the residue *n* is classified as helical; otherwise, if the distance between the amino acid *n* C-alpha and the C-alphas of amino acids *n*-2 and *n*+2 is between 5.25 and 7.75 Å, the residue *n* is classified as part of a strand; else, the amino acid *n* is considered part of a random coil. Distance thresholds were calculated and chosen from C-alpha distances found in helices and strands annotated as transmembrane found in a set of 60 non-redundant protein structures (Pellegrini-Calace et al., unpublished data).

**Assessment of effectiveness of the pore centre optimization (PoreWalker step1).**

The effectiveness of the optimization of the pore centre was assessed by monitoring the distance of the selected pore-lining residues from the pore centre. In the case of an ideal cylinder-shaped pore, as the pore vector approaches the centre of the cylinder, its distance from putative pore-lining amino acids should become constant and the corresponding standard deviation (SD) should converge to zero. However, due to the intrinsic design of the program, the process involves a stepwise increase of the distance threshold and a subsequent discontinuous increase of the number of selected pore-lining residues. Therefore, the SD was monitored using the coefficient of variation (CV, Supplementary Formula 1), which relates the SD value to the mean. The CV was calculated at each iteration of optimization for all proteins in Table 1 and normalized according to the distance threshold (CV_D_, Supplementary Formula 2) and to the number of selected pore-lining residues (CV_Naa_, Supplementary Formula 3). Trends of CV_D_ and CV_Naa_ show clearly descending values for both CVs in the channel structures, even though the channels have different shapes, size and features, and indicate that the iterative process reliably identifies optimal pore centres (Figure S1).

SD CV CV

CV = -------- (1) CV_D_ = ------- (2) CV_Naa_ = ------- (3)

M   D N_aa_

CV: coefficient of variation; CV_D_: coefficient of variation normalised by distance threshold; CV_Naa_: coefficient of variation normalised by number of selected pore-lining residues; M: mean value; D: distance threshold implemented in the selection of putative pore-lining amino acids; N_aa_: number of selected pore-lining residues.

**PoreWalker versus HOLE.**

PoreWalker and HOLE results on the 19 transmembrane proteins in the set of structures are presented in Table 1 by analysis of their diameter profiles at 1Å steps (Figures 5, S2 and S3). Pore diameter analyses performed with the two methods were in good agreement for 12 of the 19 diameter profiles, showing an R^2^ higher than 0.75. The remaining 7 profiles gave very different results, with R^2^ values very close or equal to zero (See main text). Figure 5 shows the comparison of diameters profiles and pore surfaces produced by PoreWalker and HOLE for three high R^2^ cases, the MthK calcium-gated potassium channel (1lnq, R^2^ = 0.958, Figure 3A), the MscS mechanosensitive channel (2oau, R^2^ = 0.951, Figure 3B) and the AQP0 aquaporin (1ymg, R^2^ = 0.840, Figure 3C), and three low R^2^ cases, the ASIC1 acid-sensing ion channel (2qts. R^2^ = 0.450, Figure 3d), the Amt-1 ammonium channel (2b2f, R^2^ = 0.017, Figure 3E) and the SoPIP2;1 water channel (1z98, R^2^ = 0.000, Figure 3F). The Figure clearly shows that highly correlated diameter profiles correspond to very similar identified cavities and that highest R^2^ values are observed for straight and regular pores, like the MthK and MscS channels. For more irregular structures (2qts), multiple-exited (2b2f) or “closed”(1z98) pores, although the cavities identified by HOLE and PoreWalker overlap partially or completely, the peculiar internal shape of the channels make diameter trends less correlated. It is interesting that in the case of the multiple-exited Amt-1 channel neither HOLE nor PoreWalker are able to identify the correct transmembrane pore (Figure 5A-B), although from a visual perspective PoreWalker cavity appears closer and identifies most of the relevant pore-lining residues. This could be explained by the several cavities and tunnels observed inside the protein structure, as shown by the 3D-structure of the same protein co-crystallysed with xenon (PDB code 2b2i, Figure 5A), and by the presence of a flexible external loop, highlighted in white dots in Figure 5B, which divides one of the main pore opening into multiple possible exits.

**Calculation of angle (θ) for the vertical cylindrical segment (wedge) slicing (PoreWalker step4)**

|  |                      PI * *r*  TETA = ----------  d + *r* |
| --- | --- |

*r*: average heavy atom radius (1.8 Å); d: distance between the slice pore centre (O) and an atom within the slice (X); d + *r*: approximate radius of the cylinder segment approximating one layer of atoms; PI**r*: arc A, i.e. ~half a circumference of radius *r* (~ a heavy atom); TETA: angle subtended by arc A.
