# Supplementary figures and images for: PoreWalker: A Novel Tool for the Identification and Characterization of Channels in Transmembrane Proteins from Their Three-Dimensional Structure
Source: PLoS Comput Biol. 2009 Jul 17;5(7):e1000440. doi: 10.1371/journal.pcbi.1000440 (PMC2704872; doi:10.1371/journal.pcbi.1000440)

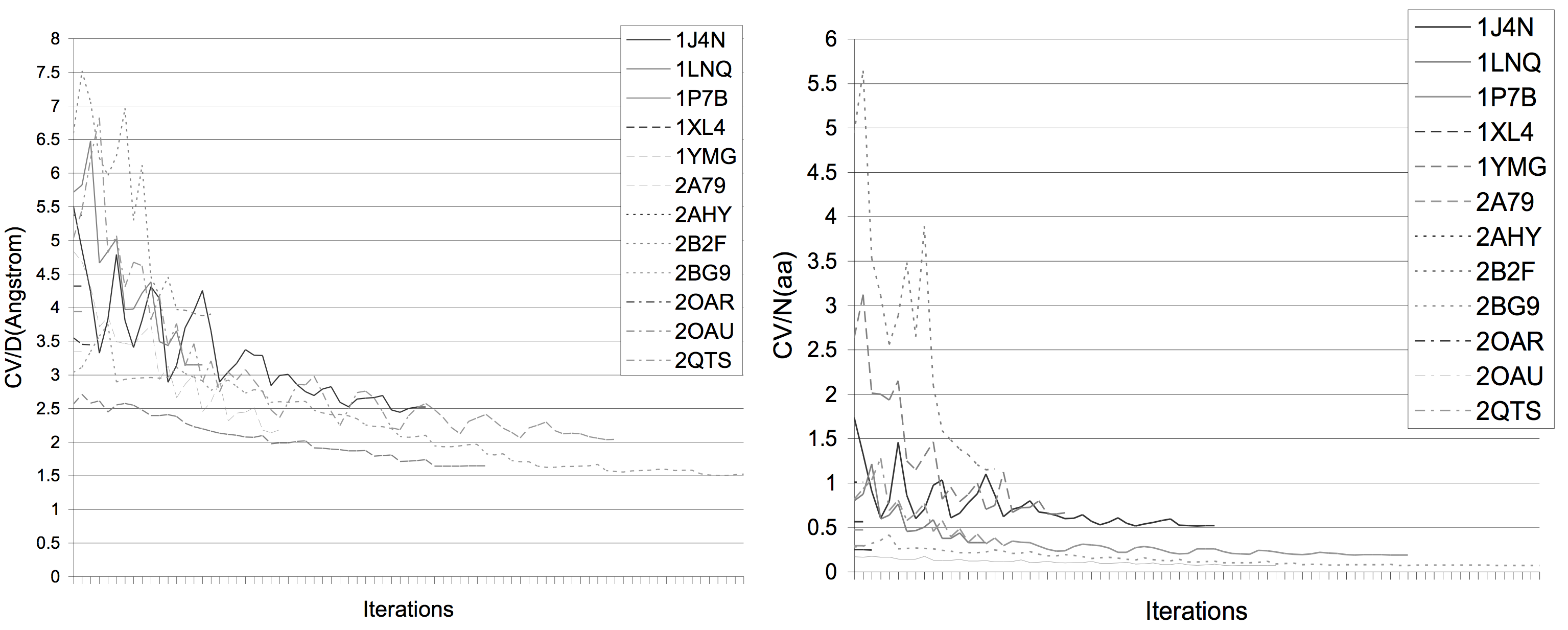

Supplement: Figure S2 — PoreWalker visual representations. Images show xz-plane sections, z-coordinate>0 only, with the x-axis corresponding to the pore axis. The remaining protein structures in Table 1 are shown. (A) KirBac3.1 potassium channel (1xl4); (B) MscS voltage-modulated mechanosensitive channel (2oar); (C) bovine aquaporin-0 (1ymg); (D) Kir3.1 prokaryotic Kir potassium channel (2qks); (E) Amt-B ammonium channel (1xqf); (F) pLGIC pentameric ligand-gated ion channel (2vl0); (G) plant SoPIP2;1 water channel (1z98). (3.62 MB TIF) [file pcbi.1000440.s002.tif]

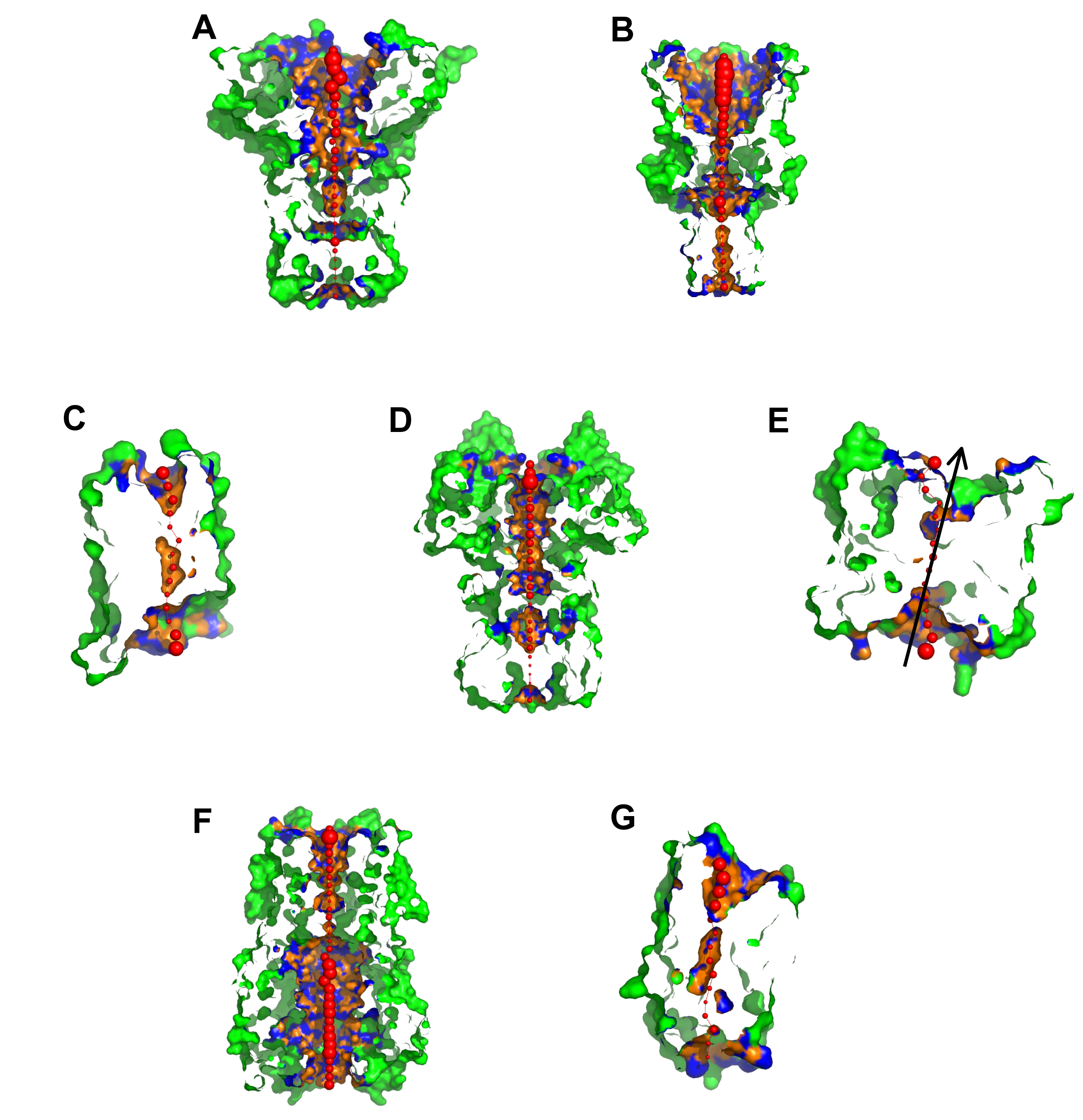

Supplement: Figure S3 — PoreWalker and HOLE diameter profiles at 1Å steps. Solid and dotted lines indicate PoreWalker and HOLE diameter profiles, respectively. (A) KirBac1.1 inward-rectifier potassium channel (1p7b, R2 = 0.918); (B) bovine aquaporin-0 (1j4n, R2 = 0.615); (C) KcsA potassium channel (1k4c, R2 = 0.740); (D) MthK calcium gated potassium channel (1lnq, R2 = 0.958); (E) KirBac3.1 inward-rectifier potassium channel (1xl4, R2 = 0.925); (F) Amt-B ammonium channel (1xqf, R2 = 0.750); (G) bovine aquaporin-0 (1ymg, R2 = 0.814); (H) plant SoPip2;1 water channel (1z98, R2 = 0.000); (I) shaker Kv1.2potassium channel (2a79, R2 = 0.583); (J) sodium-potassium channel (2b2f, R2 = 0.017); (K) Amt-1 ammonium channel (1p7b, R2 = 0.918); (L) nicotinic acetylcholine receptor (2bg9, R2 = 0.814). (0.93 MB TIF) [file pcbi.1000440.s003.tif]

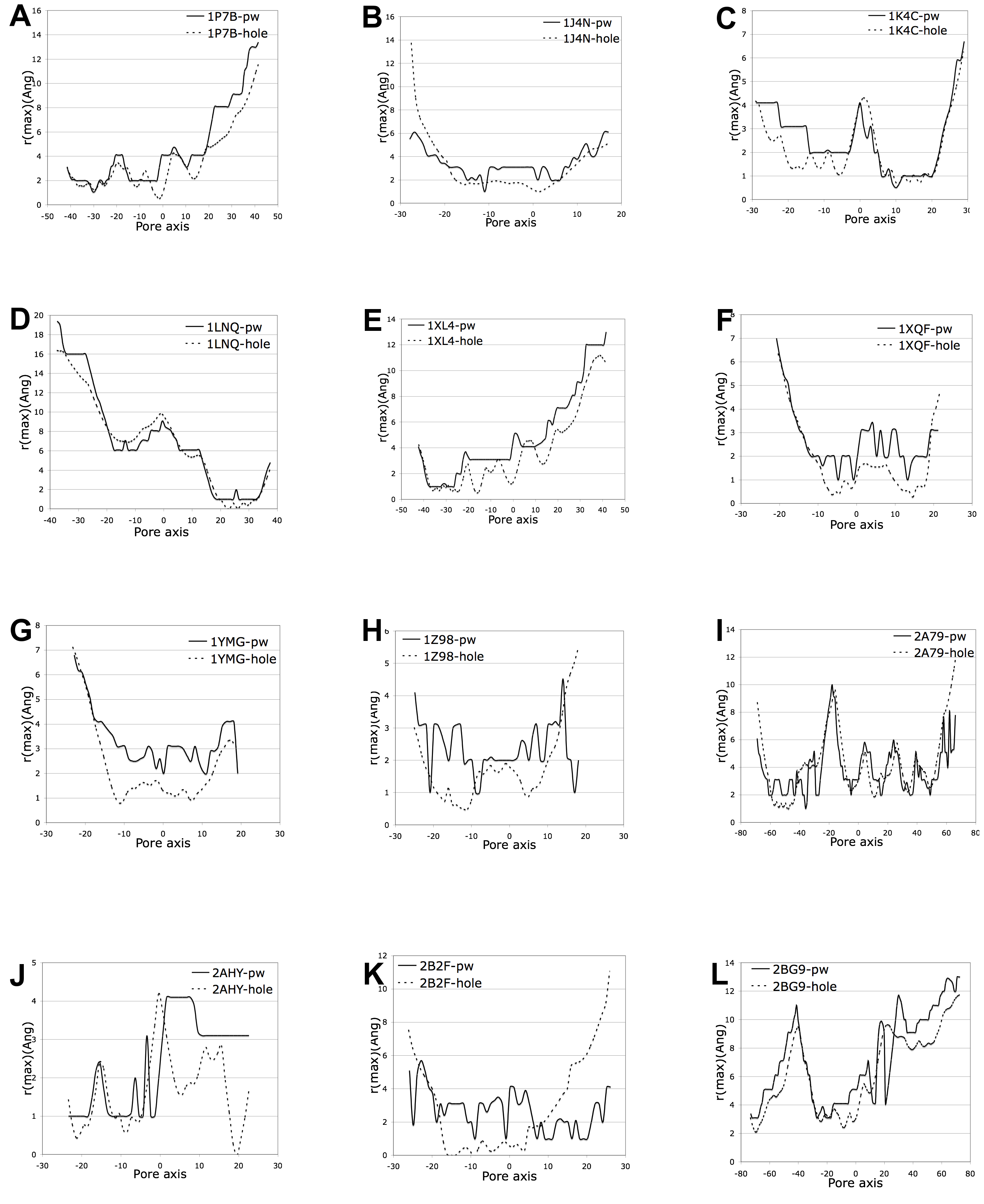

Supplement: Figure S4 — PoreWalker and HOLE diameter profiles at 1Å steps. Solid and dotted lines indicate PoreWalker and Hole diameter profiles, respectively. (A) CorA Mg2+ channel (2iub, R2 = 0.834); (B) MscL mechanosensitive channel (2oar, R2 = 0.956); (C) MscS mechanosensitive channel (2oau, R2 = 0.951); (D) Kir3.1 prokaryotic Kir potassium channel (2qks, R2 = 0.817); (E) ASIC1 acid-sensing ion channel (2qts, R2 = 0.450); (F) pLGIC pentameric ligand-gated ion channel (2vl0, R2 = 0.776); (G) SecYE-beta protein conducting channel (2yxr, R2 = 0.095). (0.63 MB TIF) [file pcbi.1000440.s004.tif]

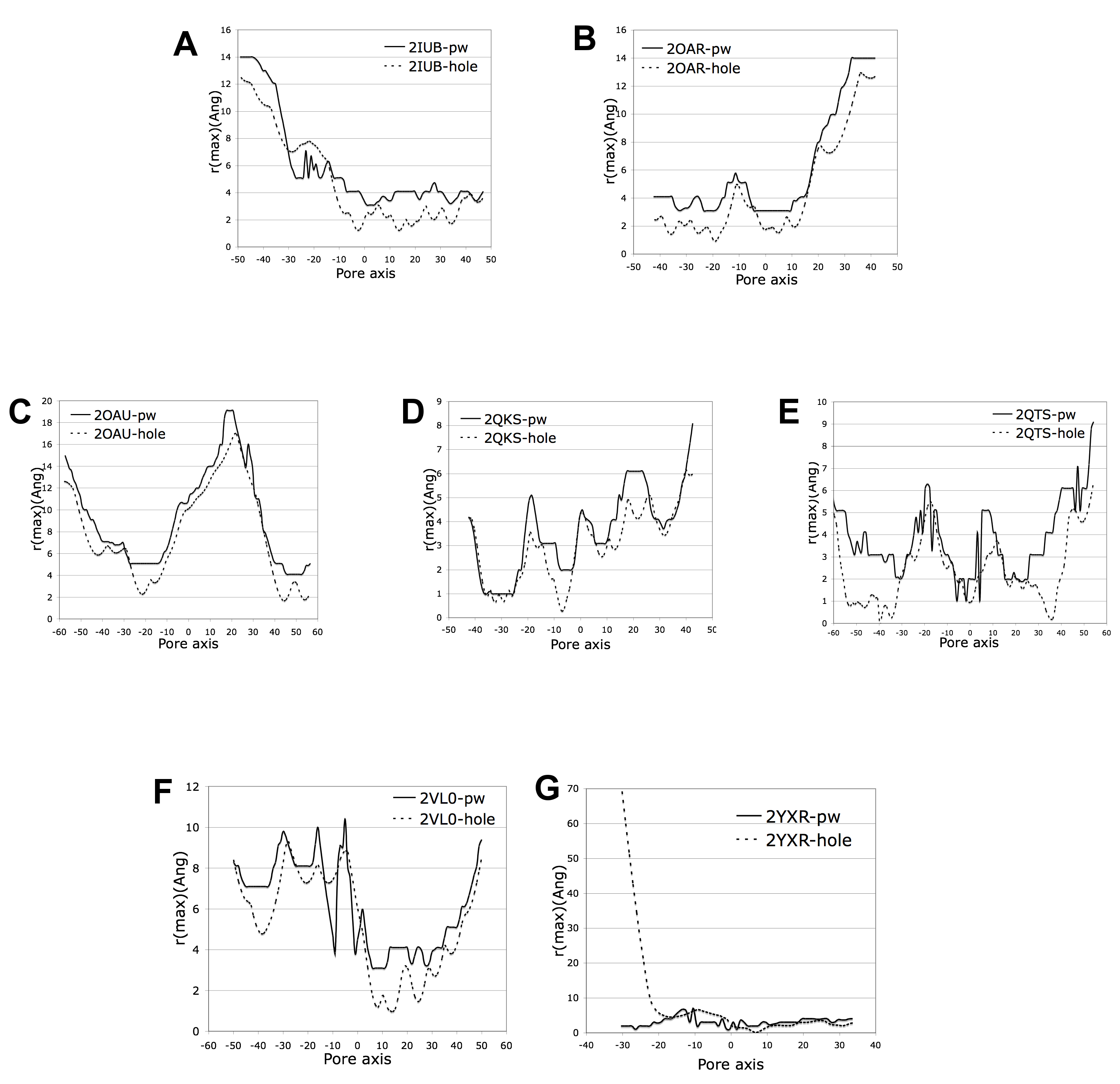

Supplement: Figure S5 — Diameter profiles versus linearity of the cavity. The correlation between R2 values of PoreWalker-HOLE diameter profiles and the percentage of number of pore centres at 1Å steps that can be fit on one or more lines with PRINCIP is shown. Each point represents one protein. The starred point indicates the only outlier point (sodium-potassium channel, PDBcode 2ahy). (0.12 MB TIF) [file pcbi.1000440.s005.tif]

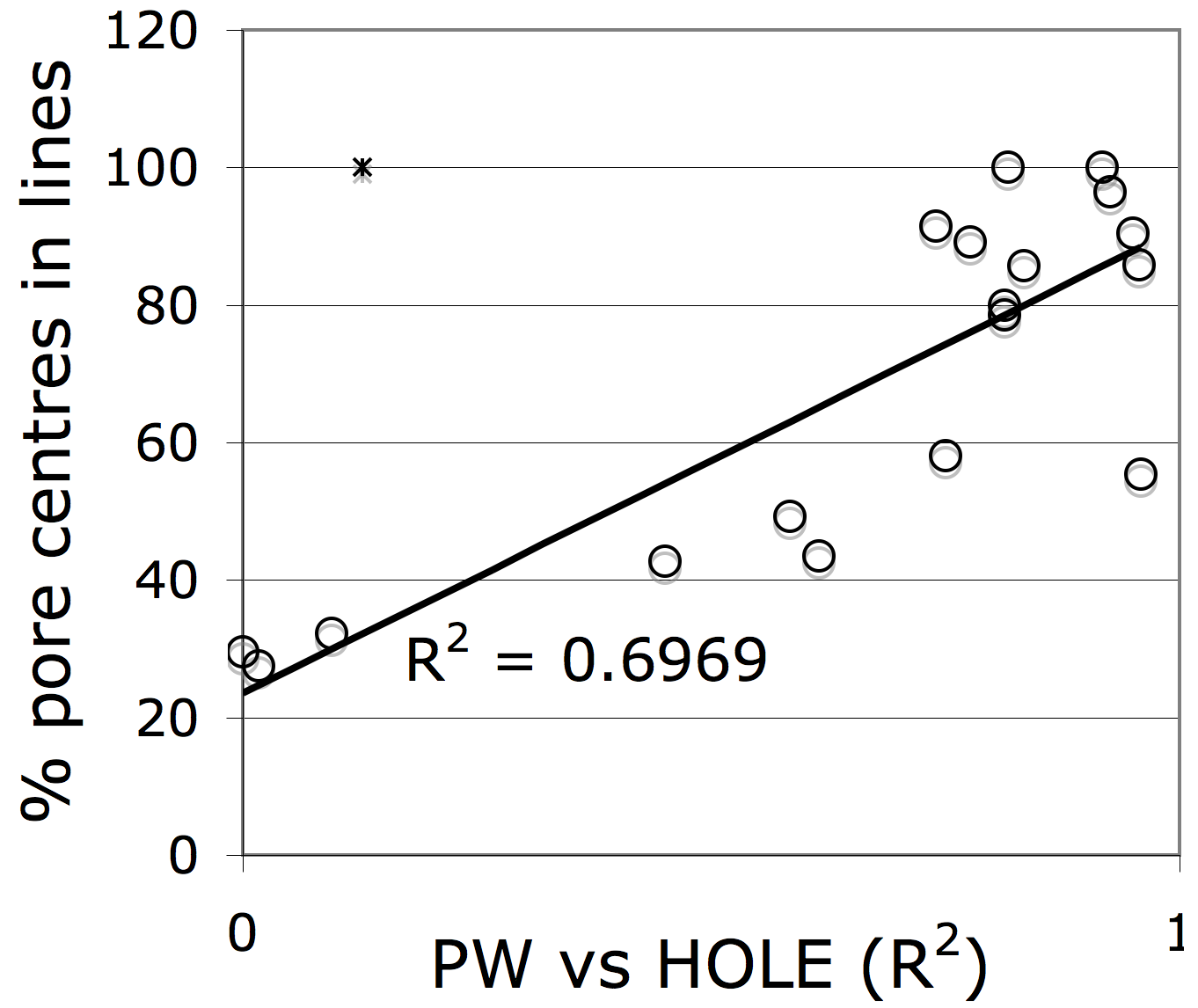

Supplement: Text S1 — Supplementary text. (0.12 MB DOC) [file pcbi.1000440.s006.tif]
